# The impact of environmental accidents on the green apparel purchase behavior of Generation Z

**DOI:** 10.3389/fpsyg.2024.1338702

**Published:** 2024-04-10

**Authors:** Lixian Liu, Wenwen Zhang, Hao Li, Zeyu Zheng

**Affiliations:** ^1^International Institute of Fashion Technology, Zhejiang Sci-Tech University, Hangzhou, China; ^2^Zhejiang Provincial Research Center for Silk and Fashion Culture, Hangzhou, China; ^3^School of Fashion Design and Engineering, Zhejiang Sci-Tech University, Hangzhou, China; ^4^Hangzhou Zhiyi Technology Co., Ltd., Hangzhou, China

**Keywords:** environmental accidents, green apparel purchase behavior, Generation Z, theory of planned behavior, norm activation theory

## Abstract

**Introduction:**

This study examines the impact of environmental accidents on Generation Z’s purchase behavior towards green apparel, integrating the Theory of Planned Behavior and the Norm Activation Theory to conceptualize how different dimensions of environmental accidents influence consumer behavior.

**Methods:**

Employing focus groups and questionnaires, this research captures data on Generation Z’s perceptions and behaviors towards green apparel in the context of environmental accidents. The collected data were analyzed using SPSS software, with structural equation modeling employed to test the research hypotheses.

**Results:**

The findings indicate that the scale (H12  = 0.545), the degree of suddenness (H18  = 0.357), nature of the effect (H14  = 0.295), and duration (H17  = 0.289) of environmental accidents significantly influence Generation Z’s awareness of consequences, behavioral attitudes, subjective norms, and perceived behavioral control regarding the purchase of green clothing. Notably, the scale of environmental accidents has a significant impact on perceived behavioral control, which in turn significantly affects Generation Z’s intention to purchase green apparel (H3  = 0.5).

**Discussion:**

This study elucidates the impact of environmental accidents on Generation Z’s green apparel purchase intentions. The findings highlight Generation Z’s environmental awareness and social responsibility, influencing their purchasing decisions. This research offers practical insights for brands to enhance green marketing strategies, focusing on product quality, transparency, and consumer education to align with Generation Z’s values and expectations. Future research should explore additional factors affecting purchase behavior and strategies to bridge the intention-behavior gap.

## 1 Introduction

In recent years, frequent environmental accidents with characteristics of environmental pollution, ecological destruction, and resource depletion caused by human activities have caused serious damage to the global environment (Prati and Zani, 2013; Gyenes and Wood, 2014). This trend has forced human civilization to shift towards responsible consumption patterns to ensure the safety and health of current and future generations (Masson-Delmotte et al., 2019). As one of the industries that have a significant impact on the environment, the textile industry has a negative impact on groundwater, air, and soil (McNeill and Venter, 2019). It is estimated that by 2030, the global environmental pressure of the textile industry will reach 27.91 million tons of emissions, consume 118 billion cubic meters of water, and generate 148 million tons of textile waste (Rausch and Kopplin, 2021). To address the environmental and social criticism facing the fashion industry, the concept of sustainability is gradually gaining attention from consumers, policymakers, and fashion retailers (McNeill and Venter, 2019). Consumer awareness of climate change and environmental issues has undergone positive changes (Black et al., 2019). This has led to steady growth in the market share of green apparel over the past decade, with the expectation that it will exceed 6% by 2026 (Statista, 2024). This growth is primarily driven by the younger generation, especially the Millennial and Generation Z, who accounted for 68% of green apparel revenue in 2022. By 2027, Generation Z is expected to account for more than one-third of the sustainable fashion market (Statista, 2023). Generation Z is intensely aware of ethics and environmental issues (Djafarova and Foots, 2022), and they are more inclined to incorporate sustainability into their lifestyle (Dabija and Brandusa, 2017; Saarelainen, 2021). To attract and retain these environmentally-conscious customers, fashion companies are striving to stimulate their green buying decisions.

However, Due to the diversity, suddenness, and randomness of environmental accidents (Ji, 2018), fashion companies cannot predict these events in advance. Therefore, they cannot adopt corresponding design, production, and marketing strategies (Kar et al., 2020). Meeting consumers’ green apparel purchase intention and promoting green purchase behavior is critical for the success of green fashion companies. Therefore, this research seek to understand Generation Z’s green apparel purchase intention and purchase behavior in the context of environmental accidents.

Currently, research on green apparel purchase behavior mainly focuses on the factors influencing green purchase intentions (Joshi and Rahman, 2015; Zhuang et al., 2021), the gap between green purchase intentions and green purchase behavior (Carrington et al., 2010; Wiederhold and Martinez, 2018; Rausch and Kopplin, 2021), and marketing strategies for green apparel (Ottman, 2017). There is still a lack of research on the specific impact of the environment on green purchase behavior. Additionally, most research on green purchasing behavior relies on single qualitative methods or literature review, which cannot accurately predict for businesses and overly relies on altruistic or self-interested consumer behavior theories, without fully considering the influence of external environment on consumer behavior, as well as the impact and mechanisms of environmental accidents on Generation Z’s green purchasing behavior.

To fill this research gap, this study adopts a combination of qualitative and quantitative research methods based on the theory of planned behavior and norm activation theory to investigate the impact of sudden environmental events on consumer purchasing behavior. a specific measurement framework is constructed to provide empirical research support for fashion companies’ decision-making. The specific research objectives of this study include:

1. Constructing evaluation dimensions for environmental accidents,
2. Developing a theoretical framework for consumer green apparel purchasing behavior in the context of sudden environmental events, and
3. Providing decision-making recommendations for businesses to address green apparel purchasing behavior in the face of environmental accidents.

In this study, focus groups are used to divide the dimensions of environmental accidents, and then a questionnaire survey is conducted to conceptualize the dimensions of environmental accidents that influence Generation Z’s green apparel purchasing behavior. The theoretical hypotheses are empirically tested using SPSS software and AMOS. This paper is mainly divided into several sections, including Introduction, Literature Background, Theoretical Underpinnings and Hypotheses Development, Methodology, Results, Discussion and Implications, Limitations and Future Research, aiming to provide a comprehensive discussion and analysis through clear reasoning, and to provide strong theoretical and decision-making support to improve the current development status of the eco-fashion industry and assist businesses in dealing with environmental accidents.

## 2 Literature background

### 2.1 Environmental accident

In The National Contingency Plan of environmental accident, environmental accident refers to an event caused by human activities that result in adverse consequences such as environmental pollution, ecological damage, and resource depletion, causing severe environmental harm. Examples of such events include oil spills, chemical leaks, and nuclear accidents, which can abruptly cause or potentially cause a decrease in environmental quality, endanger public health and property safety, cause ecological damage, or have significant social impacts. These events require urgent measures to be taken to address them (The National Contingency Plan for Environmental Accidents, 2014).

Current research on environmental accidents covers various topics, including analyzing their spatial and temporal characteristics (Li et al., 2008), exploring influencing factors (Li and Hu, 2020; Guo et al., 2021; Wan et al., 2021), occurrence mechanisms (Wang et al., 2010), risk assessment (Liao et al., 2012; Wang et al., 2020), response measures (Pawełczyk et al., 2018), and their relationship with economic development (Li et al., 2008; Wang et al., 2020). Scholars and experts have approached the subject from different perspectives, digging deeper into environmental accidents’ root causes and characteristics.

From the perspective of research content, the spatiotemporal characteristics and influencing factors of environmental accidents are two important research directions. The relevant research on the spatial and temporal characteristics of environmental accidents mainly focuses on the evolution of environmental accidents in time and space. It discusses the laws of their formation and development to provide a scientific basis for predicting and preventing environmental accidents (Li et al., 2008). The relevant research on the influencing factors of environmental accidents mainly targets various complex factors in the formation, development, and progress of environmental accidents. It analyses the role and influence of these factors to guide the formulation of effective coping strategies and measures (Wang et al., 2010; Li and Hu, 2020; Guo et al., 2021; Wan et al., 2021).

The occurrence mechanism of environmental accidents and risk assessment are also popular topics for research. By studying the occurrence mechanism of environmental accidents, we can better grasp the nature and characteristics of environmental accidents and provide a scientific basis for response and prevention (Wang et al., 2010, 2020). Similarly, risk assessment, as an essential means of responding to environmental accidents, plays a vital role in early warning systems and mitigating the impact of environmental accidents (Liao et al., 2012). Through the identification, analysis, and evaluation of risk factors, the risk degree of environmental accidents can be evaluated more scientifically and objectively to provide a reference for formulating effective countermeasures (Pawełczyk et al., 2018).

The study of environmental accidents is a complex and vital field that requires interdisciplinary cross-border cooperation. This research should help us face future environmental challenges, establish an environmental accident warning system, and improve the environmental accident response capacity.

### 2.2 Generation Z green apparel purchase behavior

Green apparel purchase behavior refers to consumers considering the environmental impact of apparel and purchasing environmentally friendly and sustainable apparel. This behavior is usually influenced by factors such as consumer awareness of environmental issues, environmental consciousness, and environmental knowledge (Khare, 2023). The apparel industry is recognized as one of the world’s largest contributors to various social and environmental problems (McNeill and Venter, 2019). It accounts for 10% of global carbon emissions, ranking as the second most polluting sector globally (Dhir et al., 2021).

To mitigate the negative consequences caused by the fashion industry, a potential solution is to depart from the prevailing “fast fashion” model that has long dominated the industry, and embrace green apparel instead (Khare, 2023). Green apparel significantly reduces its environmental impact through sustainable manufacturing practices. Typically, such apparel is made from natural fibers without the use of harmful chemicals (Chan and Wong, 2012; Cowan and Kinley, 2014; Harris and Helen Roby, 2016). Several characteristics define green apparel, including the use of recycled materials, longevity in style as opposed to quickly outdated trends, organic cultivation of natural fibers, low or no-dye processing, and environmentally friendly labeling or packaging (Kim and Damhorst, 1998; Connell, 2010).

Research has shown that consumers hold a positive attitude towards eco-friendly clothing (Ellen et al., 1991; Liu et al., 2012), especially Generation Z consumers (Statista, 2023). Gen Z was born during the rise of the digital society and grew up during the digital technology boom. They are the first generation whose life experiences are fully embedded in the digital society (Törőcsik et al., 2014; Turner, 2015; Dolot, 2018; Dimock, 2019). Gen Z tends to show high participation and proficiency in information acquisition and dissemination when facing environmental accidents. Therefore, studying Gen Z’s green apparel purchase behavior in the context of environmental accidents becomes particularly important.

Previous research on Gen Z’s apparel purchase behavior mainly focused on their individual characteristics, consumer beliefs, and their causes (Chaturvedi et al., 2020; McCoy et al., 2021; Arora and Manchanda, 2022) while neglecting the transmission path of green awareness and emotional impact in the environmental accident for Gen Z consumers in the internet environment. Behavioral theory-based research can explore the transmission path of the impact of environmental accidents on green awareness and emotions of Gen Z and provide a more comprehensive understanding of the influence of self-interest and altruism on Gen Z’s green apparel purchase behavior. Therefore, this study conceptualizes the consumer’s construction of awareness of environmental accidents and empirically determines the mechanism by which environmental accidents affect Gen Z’ green apparel purchase behavior.

## 3 Theoretical underpinnings and hypotheses development

### 3.1 Theory of planned behavior and norm activation theory

#### 3.1.1 Theory of planned behavior (TPB)

The Theory of Planned Behavior (TPB), proposed by Icek Ajzen in 1985, is currently one of the most potent theories for predicting behavior and has been widely used in research on environmental behavior (Joshi and Rahman, 2015). The TPB argues that an individuals behavior results from rational thought and planning. It comprises three core concepts: behavioral attitudes, subjective norms, and perceived behavioral control. Behavioral attitudes refer to an individuals evaluation of a particular behavior, including whether the behavior is good or bad, favorable or unfavorable. Subjective norms refer to individual expectations and pressures for others and the degree of attention paid to others evaluations. Perceived behavioral control is the degree of confidence that they can control their behavior (Ajzen, 1985, 1991; Fishbein and Ajzen, 2011).

**Figure 1:**
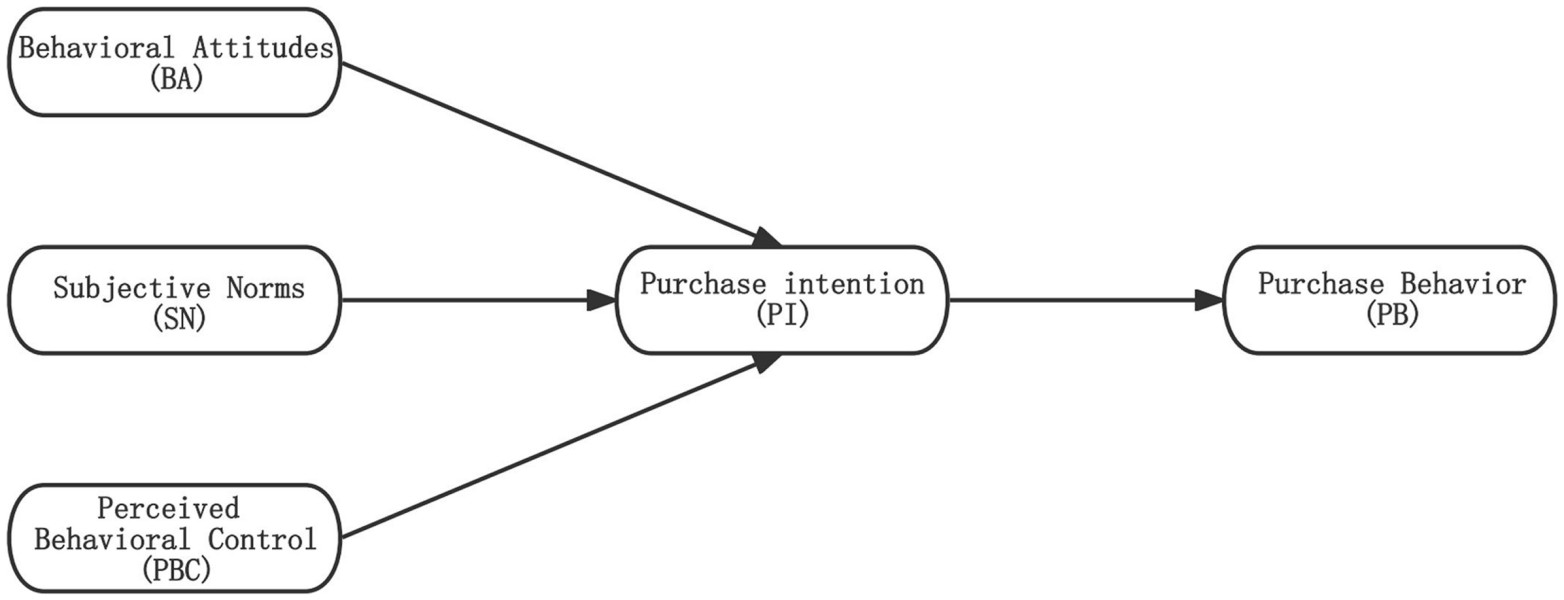
The theory of planned behavior framework.

According to the TPB, these three core concepts determine an individual’s purchase intentions. Behavioral attitudes, subjective norms, and perceived behavioral control influence their intentions toward particular behaviors, and purchase intention is the critical determinant of whether an individual will take action (Figure 1).

In relevant research on the TPB, Kang et al. (2013) examined sustainable textile apparel purchase behavior among young consumers in China, the United States, and South Korea. They found that behavioral attitudes, perceived behavioral control, and subjective norms positively influenced consumers’ purchase intention. Liu (2022) discovered that the attitudes of Generation Z directly impacted their purchase intention when buying sustainable clothing. Moreover, Rausch and Kopplin (2021) study revealed that consumers’ attitudes towards sustainable clothing and subjective norms significantly influenced purchase intention. At the same time, purchase intention also substantially impacts the purchase behavior of Generation Z consumers. Accordingly, this research proposes the following hypotheses in the context of green apparel purchase:

*H1*: In the green apparel purchase behavior of Generation Z, behavioral attitudes have a positive impact on purchase intention.

*H2*: In the green apparel purchase behavior of Generation Z, subjective norms have a positive impact on purchase intention.

*H3*: In the green apparel purchase behavior of Generation Z, perceived behavioral control has a positive influence on purchase intention.

*H4*: In the green apparel purchase behavior of Generation Z, purchase intention has a positive influence on purchase behavior.

#### 3.1.2 Norm activation theory (NAT)

**Figure 2:**
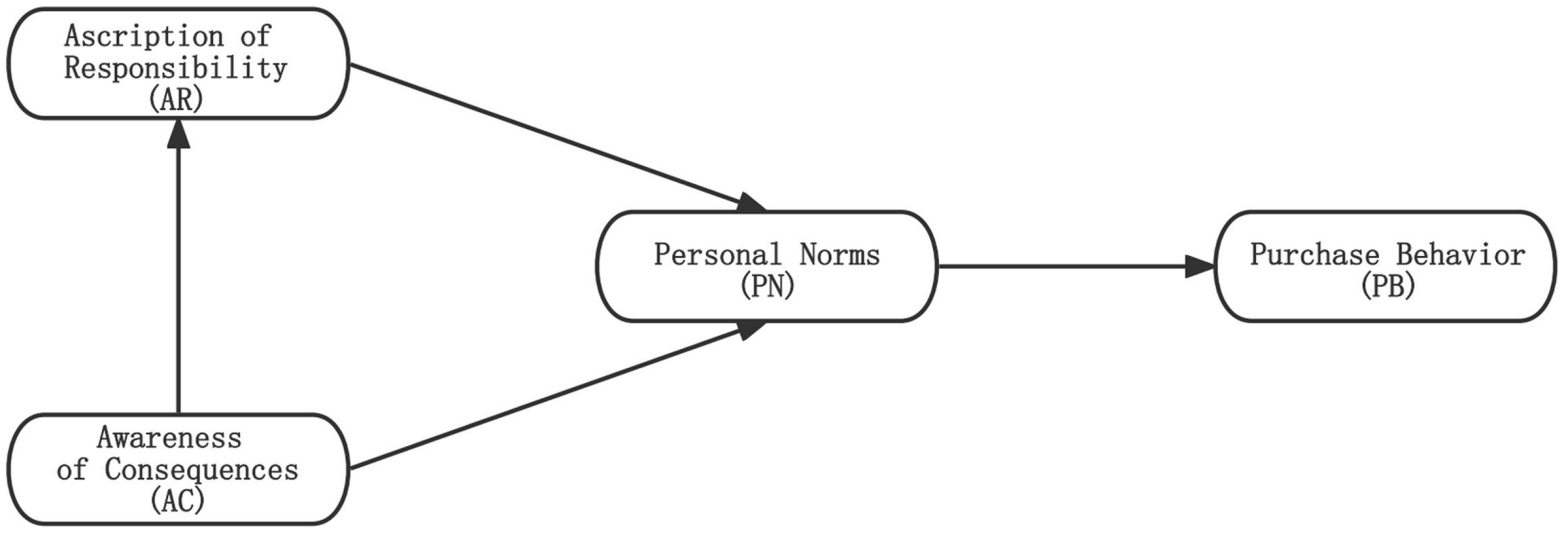
The norm activation theory framework.

Norm Activation Theory (NAT) is a behavioral theory proposed by Schwartz to explain and predict the pro-social behaviors of individuals (Schwartz, 1977). In the application of specification activation theory to present a series of pro-social and environmental behaviors, Steg and De Groot (2010) found the relationship between awareness of consequences, ascription of responsibility, personal norms, and purchase behavior to be a chain-mediated model, meaning that consequence perception activates individual norms through responsibility attribution, which in turn leads to the occurrence of individual pro-social and pro-environmental intentions. NAT states that individuals’ awareness of potential harmful consequences and their sense of responsibility trigger personal norms, determining engagement in specific behaviors to prevent unfavorable outcomes (Figure 2).

NAT is a theory that explains how individuals perceive and respond to norms in social interactions. It emphasizes the channels through which normative information is activated and the processes through which individuals respond to norms. This theory has extensive application value in studying green purchase behaviors, focusing on analyzing altruistic factors to understand how individuals engage in green consumption influenced by others’ environmental behaviors and social expectations (Han, 2014; Zhang et al., 2017; Confente and Scarpi, 2021).

In studies on the NAT, the awareness of consequences has been identified as a critical variable for predicting environmentally friendly intentions. It positively influences personal norms and the ascription of responsibility (Schwartz, 1977). The awareness of consequences regarding global and local environmental issues can lead to pro-environmental behavior through the mediating effects of the ascription of responsibility and personal norms (Schultz et al., 2005; Milfont et al., 2010). Furthermore, personal norms may also be influenced by the ascription of responsibility. Personal norms refer to internal values or behavioral guidelines that influence individuals’ choices in specific situations. In contrast, the ascription of responsibility refers to individuals’ perceptions and ascription of responsibility for an event. When individuals attribute blame to themselves, they are more likely to take responsibility and engage in corresponding actions to address the issue. For example, a study by Ebreo et al. (2003) found that the ascription of responsibility significantly predicted environmentally related behaviors, as individuals who blamed themselves were more inclined to engage in waste reduction behaviors. Personal norms may also influence purchase behavior. Personal norms can impact the willingness of Generation Z to purchase green apparel, thereby ultimately affecting consumer purchase behavior (Chaturvedi et al., 2020). Based on these findings, this study proposes the following hypotheses in the context of green apparel purchases:

*H5*: In the green apparel purchase behavior of Generation Z, awareness of consequences has a positive impact on the ascription of responsibility.

*H6*: In the green apparel purchase behavior of Generation Z, awareness of consequences has a positive influence on personal norms.

*H7*: In the green apparel purchase behavior of Generation Z, the ascription of responsibility has a positive impact on personal norms.

*H8*: In the green apparel purchase behavior of Generation Z, personal norms have a positive influence on purchase behavior.

### 3.2 An integrated theoretical framework based on the TPB and NAT

**Figure 3:**
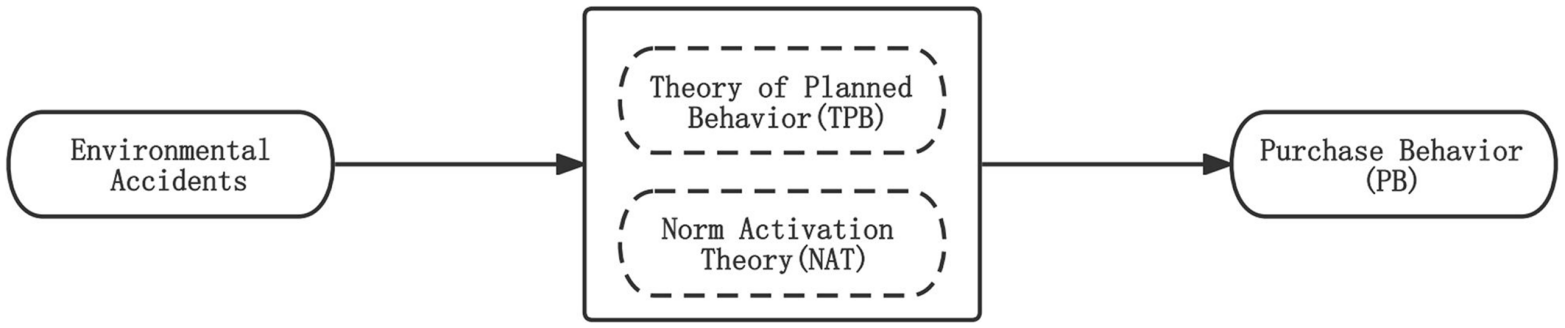
Integrated theoretical framework based on the theory of planned behavior and norm activation theory.

The TPB takes egoism as a starting point, with the principle of maximizing benefits as the decision-making criterion. In contrast, the NAT emphasizes altruistic tendencies and moral obligations as driving forces (Hofenk et al., 2010; Park and Ha, 2014; Shin et al., 2018). According to Lao (2014), consumers’ purchase behavior for green products combines both altruistic and self-interested considerations. Environmental accidents can trigger a stronger sense of moral responsibility and lead to changes in green purchasing behavior. By incorporating NAT into the framework, the study can better explain these changes. However, studying consumers’ green apparel purchase behavior solely from a self-interest perspective would overlook the impact of altruistic motives. After conducting a comparative empirical study on TPB and NAT, Teisl et al. (2009) found that integrating the two models into the same theoretical framework enhances the predictive and explanatory power of the models for environmental behavior (Figure 3).

In the integrated theoretical framework, subjective norms can influence personal norms. Subjective norms refer to the perceptions of influential individuals and groups with relevant opinions and evaluations of one’s behavior. Individuals can use these opinions and perceptions to judge the social correctness of their behavior, thereby guiding consumers to assess the consistency of their behavior with their intrinsic self-expectations and values. Currently, in research on TPB and NAT, Kim and Seock (2019) found that personal norms significantly influence consumers’ purchase intentions for environmentally friendly products. The perceived subjective norms can indirectly influence purchase intentions through personal norms, and the awareness of consequences may influence behavioral attitudes. Behavioral attitudes represent consumers’ opinions and evaluations of their purchasing behavior, reflecting their cognitive understanding of their purchasing behavior. The awareness of consequences reflects consumers’ cognition of the impacts of their behavior outcomes and, therefore, naturally influences their attitude towards engaging in a specific behavior. For example, Han (2015) found that individuals exhibit a more positive attitude towards choosing to stay in a green hotel when they perceive the adverse environmental effects of conventional accommodations. Liang and Cheng (2020) also pointed out that most Chinese consumers rarely consider the adverse environmental impacts of clothing when making purchases, leading to their negative and uninformed attitudes toward purchasing green clothing. Based on this, this study proposes the following hypotheses in the context of green apparel purchase behavior among Generation Z:

*H9*: The awareness of consequences positively influences behavioral attitudes In the green apparel purchase behavior of Generation Z.

*H10*: Subjective norms positively influence individual norms In the green apparel purchase behavior of Generation Z.

In conclusion, the combination of TPB and NAT integrates self-interest and altruism, and it is widely applied in theoretical research on green consumption behavior. TPB helps us understand the influence of intentions and subjective norms on purchase behavior in environmental accidents. In contrast, NAT helps us understand the influence of activated moral norms on behavior under the impact of environmental accidents. By combining these theories, this study can explain and predict consumers’ green purchase behavior, contributing to a better understanding of the green economy and sustainable development goals. It provides decision-making support for businesses and governments in the context of green apparel purchase behavior among Generation Z.

### 3.3 Focus group

Currently, research on environmental accidents mainly focuses on the degree of suddenness (such as unpredictability and instantaneous occurrence), the nature of effects (destructiveness and societal harm), and the duration of the events (Wang et al., 2010, 2020). Scholars consider the degree of suddenness to describe the unexpectedness of environmental accidents. The nature of effects refers to the definition of the impact or consequences of environmental accidents and serves as an essential reference for defining such events. Duration refers to the length of time from the occurrence of an event to its end, including the development, evolution, and lasting effects of the event. The duration of environmental accidents is one of the essential indicators to measure their impact and difficulty in response.

Additionally, scholars have also studied the scope of impact, the domains of occurrence, and the causes of events (WHO, 1998; Prati and Zani, 2013; Steinhauser et al., 2014; Ding et al., 2015; Marta-Almeida et al., 2016; Wang et al., 2020), reflecting the scale of different sudden events (in terms of affected area or range). Therefore, this study believes that the dimensions of research on environmental accidents primarily consist of the degree of suddenness, the nature of effects, duration, and magnitude of impact. Since this summarization has yet to be systematically expounded and validated, in this study, data collection was conducted through focus groups (FG). A combined qualitative and quantitative approach was used to determine the evaluation dimensions of sudden environmental accidents.

The study included 24 Gen Z participants from Chinese universities, consisting of 14 undergraduates and 10 graduates students covering various academic backgrounds. Participants were divided into three groups (A, B, and C) of eight members each. Each group was facilitated by a trained moderator who conducted semi-structured discussions around the topic “What factors do you primarily consider when evaluating and researching environmental accidents?.” Participants were informed about using audio and video recording devices, and their consent was obtained. To quickly focus participants’ attention on environmental accidents, at the beginning of the focus group discussions, the moderator introduced two representative environmental accidents – the leaks in the Nord Stream pipelines and the Chernobyl nuclear power plant accident. These two events belong to different types of environmental accidents and are both sudden, unpredictable, and hazardous incidents caused by human activities.

Based on the literature review mentioned earlier and the degree of suddenness, magnitude of impact, nature of effects, and duration of environmental accidents, an interview guide for the focus group experiment was developed. Some of the questions in the interview guide were: (1) From which channels do you learn about environmental accidents? (2) Has this event affected your purchase behavior for green apparel? What impacts did it have? (3) What factors do you consider when assessing the degree of environmental accidents? (4) What factors do you consider when assessing the duration of environmental accidents? A total of 275 min of interview recordings were collected from the three FG sessions, which were transcribed into text files immediately after each discussion. In total, the transcription yielded 91,400 words.

This study used the semantic analysis method (Landauer et al., 1998) to analyze the interview transcripts. The purpose was to gain insights into environmental accidents and measure their various dimensions. The analysis process involved several stages.

In the data preprocessing stage (Famili et al., 1997), the interview transcripts underwent several important steps to clean and prepare the text for analysis. Initially, word segmentation was performed to split the text into individual words or tokens. This step is crucial as it helps identify meaningful units for further analysis. Stop words, which are commonly used words that do not contribute much to the overall meaning, were then removed to reduce noise and improve analysis efficiency. Lemmatization was also applied to reduce words to their base or dictionary forms, allowing for effective comparison and analysis. Furthermore, named entity recognition techniques were employed to identify and categorize specific information such as names, locations, organizations, and events. These preprocessing steps helped refine the data and enhance the quality of subsequent analysis.

Next, the parsing tree construction phase was conducted. The Stanford Parser was used in this study to build fully annotated parsing trees, which represent the syntactic structure of the sentences in a hierarchical manner. The parsing trees provide insights into the relationship between words and phrases, enabling a more in-depth understanding of the textual data. Upon the parsing tree, the NLTK library (Loper and Bird, 2002) was employed to annotate the parsing tree, enabling understanding of the semantic structure and meaning of the sentences. This step is crucial for gaining deeper insights into the underlying ideas in the text.

Subsequently, a TF-IDF modeling approach (Aizawa, 2003) was employed for feature extraction. TF-IDF stands for Term Frequency-Inverse Document Frequency and is a commonly used statistical method for assessing the importance of terms in a corpus of documents. By transforming the unstructured text data into structured numerical variables, this approach allowed for a robust analysis of the interview transcripts. The TF-IDF modeling approach provided insights into the relative significance of different terms within the data, contributing to a comprehensive understanding of the environmental accidents discussed in the interviews.

Finally, the output of the TF-IDF model was analyzed. Through this analysis, it was determined that there are eight dimensions to measure environmental accidents. These dimensions include suddenness (the abruptness of the accident), duration (the length of time the accident lasted), nature of effects (the type of impact resulted from the accident), event scale (the size and magnitude of the accident), causes of events (the factors that contributed to the accident occurrence), extent of impact (the reach and severity of the accident’s consequences), outcomes (the results or effects of the accident), and location (the specific area or place where the accident took place). These dimensions provided a comprehensive framework for understanding and assessing environmental accidents based on the interview data.

Based on the FG experiments and the results of semantic analysis, this study developed 117 items for evaluating the dimensions of environmental accidents using the card sorting method. Six experts (including two university teachers, two business managers, one design director, and one fashion buyer) assessed the content validity of these items (Churchill, 1979). After assessment, 26 unclear, unnecessary, or redundant items were removed, resulting in a final set of 91 items. These items covered four dimensions: event scale (28 items), degree of suddenness (19 items), duration (23 items), and nature of effects (21 items). Environmental accidents referred to adverse consequences caused by human activities, such as environmental pollution, ecological destruction, and resource depletion, which have severe impacts on the environment. Refer to Table 1 for detailed information.

**Table 1:** Collation of expert opinion interview results.

Dimension	Description of content	Number of projects
Event scale	The scale of the impact of an environmental accident refers to the extent of its influence, including the geographical scope, scale of the affected population, and industry scope.	28
The degree of suddenness	The suddenness of an environmental accident, including its unpredictability and the degree of unpredictability, refers to the extent to which an environmental accident can be foreseen.	19
Duration	The duration of an environmental accident refers to the length of time it takes for an environmental accident to evolve from its occurrence to its resolution or to its becoming manageable.	23
Nature of Effects	The effect nature is caused by an environmental accident, whether positive or negative, constructive or destructive.	21

### 3.4 The dimensions of environmental accident and their variable relationships

Based on the results of the focus mentioned above group discussions, this study will analyze environmental accidents from four dimensions: event scale, the degree of suddenness, duration, and nature of effects.

Van Bavel et al. (2020) discovered a relationship between the scale of environmental accidents and subjective norms. During large-scale public health crises, individuals tend to follow government instructions to mitigate virus transmission risks. Increased public attention and discussion create consensus and social pressure, influencing individuals to adopt behavior aligned with the event’s scale. Consequently, as the scale of environmental accidents magnifies, Gen Z consumers are more likely to be swayed by social consensus and inclined to purchase green apparel in support of sustainability and environmental protection. The study also suggests that the scale of environmental accidents impacts individuals’ perception of behavioral control. As the pandemic intensifies, people feel a heightened sense of control over pandemic-related information. Consequently, in the context of purchasing green apparel, expanding environmental accidents bolster individuals’ perception of environmental concerns and sustainability. This heightened perception of behavioral control prompts Gen Z consumers to consider the environmental impact of their purchase decisions and motivate them to choose green apparel. Therefore, the following hypotheses are proposed:

*H11*: The scale of environmental accidents has a positive impact on subjective norms in Gen Z's green apparel purchase behavior.

*H12*: The scale of environmental accidents has a positive impact on the perception of behavioral control in Gen Z's green apparel purchase behavior.

Environmental accidents may affect subjective norms as situational cues can influence attitudes and behaviors. The nature of the event’s effects may alter an individual’s context, intensifying their sense of social responsibility and moral obligation. Behavior attitudes can be influenced by the nature and scale of the events, causing individuals to adjust their attitudes and behavioral intentions based on the nature and impact of the event. For example, after the 9/11 attacks, Americans’ attitudes toward national security and counterterrorism significantly changed, and the increased social pressure motivated individuals to change their behavioral intentions (Pyszczynski et al., 2003). Therefore, as the scale of environmental accidents increases, Gen Z consumers are more likely to be influenced by the event and social pressure, thereby strengthening their sense of social responsibility and moral obligation. This may lead them to choose to purchase more green apparel to support sustainable development goals. Based on this, the following hypotheses are proposed in the context of purchasing green apparel:

*H13*: The nature of the effects of environmental accidents has a positive impact on behavior attitudes in Gen Z’s green apparel purchase behavior.

*H14*: The nature of the effects of environmental accidents has a positive impact on subjective norms in Gen Z’s green apparel purchase behavior.

The duration of environmental accidents affects the perception of consequences, subjective norms, and behavioral attitudes. As the duration of a sudden environmental event increases, prolonged stress can impact an individual’s perception of the environment, making them more attentive and sensitive to potential consequences. Avraham Bleich et al. (2003) suggest that longer-lasting environmental accidents result in a more significant perception of consequences and a lengthier recovery process, leading Gen Z to be more concerned and sensitive to environmental issues and impacts. Therefore, they may be more inclined to purchase green apparel to reduce adverse environmental effects. Additionally, Bhattacherjee and Sanford (2006) found that during an economic crisis, participants feel increasing social pressure over time to make donations, leading them to take actions that align with subjective norms to obtain moral recognition and a sense of self-worth. This may reinforce Gen Z’s subjective norms perception towards environmental protection, as they may acknowledge the importance of environmentally friendly behaviors and comply with societal moral standards by choosing to purchase green apparel. Furthermore, prolonged sudden events often gain extensive attention, and media and social platforms frequently report updates and related information. Schultz and Stone (1994) suggested that people who receive long-term environmental education tend to demonstrate more environmentally friendly behaviors and attitudes. As the duration of environmental accidents increases, Gen Z may show a higher level of attention and awareness towards environmental issues, leading to a more positive behavioral attitude. They may be more willing to support green apparel brands and purchase sustainable clothing products. Based on these observations, the following hypotheses are proposed in the context of purchasing green apparel:

*H15*: The duration of environmental accidents has a positive impact on the perception of consequences in Gen Z’s green apparel purchase behavior.

*H16*: The duration of environmental accidents has a positive impact on behavioral attitudes in Gen Z’s green apparel purchase behavior.

*H17*: The duration of environmental accidents has a positive impact on subjective norms in Gen Z’s green apparel purchase behavior.

Environmental accidents’ suddenness limits information acquisition and understanding. Psychological responses, information retrieval, emotions, and cognitive factors, as well as social factors, can influence their perception of consequences. Mileti (1999) stated that the degree of environmental accidents’ suddenness affects the perception of consequences. Predictability of earthquakes leads to preventative measures; otherwise, people may avoid action. Environmental accidents often draw people’s attention and alertness. When Gen Z recognizes the potential consequences of these sudden events, they may become more attentive and sensitive toward environmental issues and be motivated to take action, such as purchasing green apparel to reduce negative environmental impacts. The degree of environmental accidents’ suddenness may also influence behavioral attitudes. Environmental accidents are often perceived as high risk, and this risk perception can influence people’s evaluation of consequences and behavioral attitudes. They may become more cautious, taking precautionary measures or avoiding potential risk areas. Brewer et al. (2007) found that when people perceive influenza outbreaks as predictable, they are more willing to take preventive measures; otherwise, if they lack precautionary knowledge, they are unlikely to take action. This illustrates how the sudden nature of environmental accidents makes people more susceptible to perceiving risks and threats, which may enhance Gen Z’s attention to environmental protection issues and lead to a more positive behavioral attitude. They may be more willing to choose to purchase green apparel to reduce environmental impacts. Based on this, the following hypotheses are proposed in the context of purchasing green apparel:

*H18*: The degree of environmental accidents’ suddenness has a positive impact on the perception of consequences in Gen Z’s green apparel purchase behavior.

*H19*: The degree of environmental accidents’ suddenness has a positive impact on behavioral attitudes in Gen Z’s green apparel purchase behavior.

**Figure 4:**
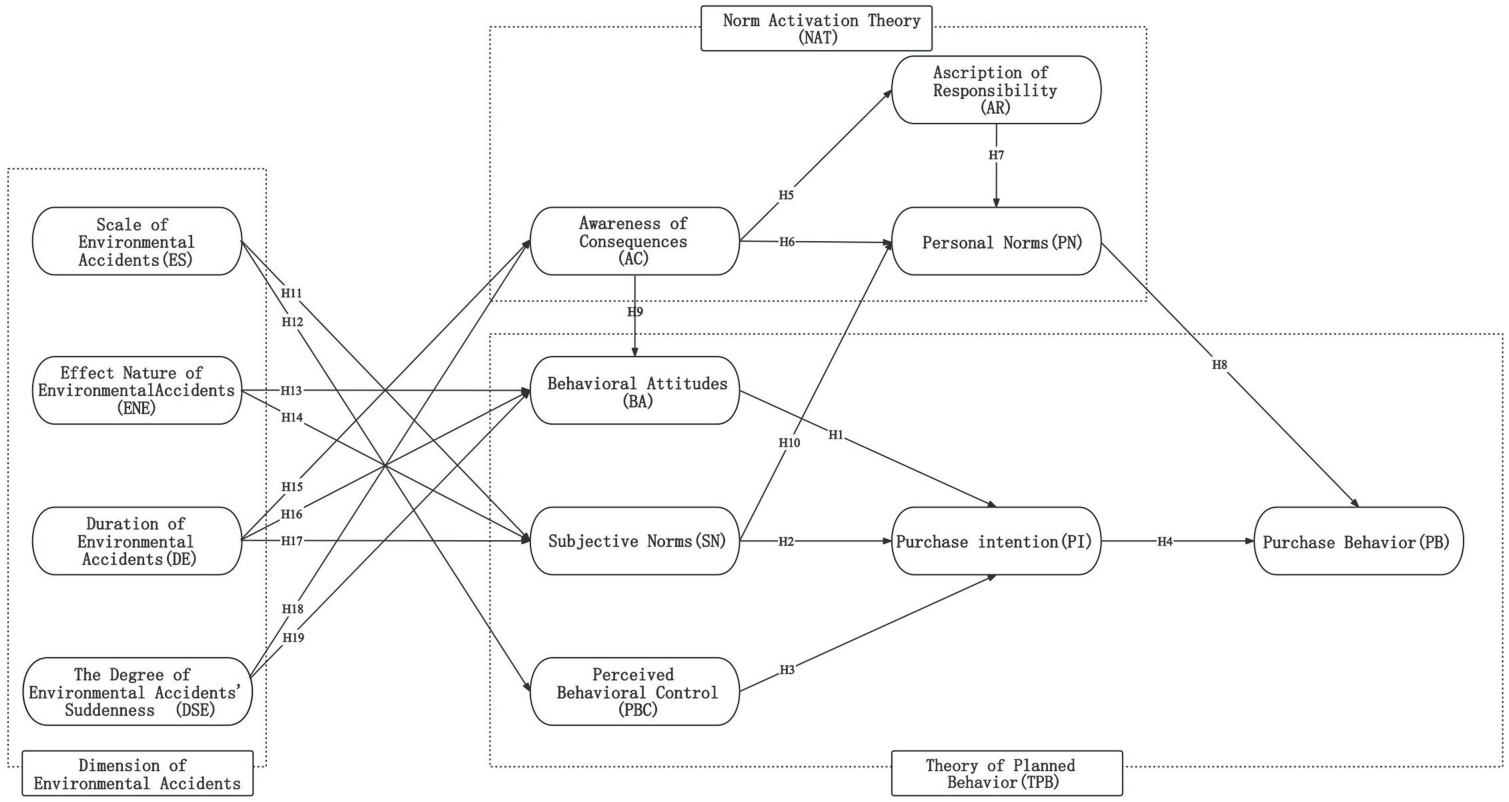
Theoretical framework of green apparel purchase behavior under environmental accidents.

Based on the theoretical analysis and research hypotheses mentioned above, the relationships between variables in this study’s theoretical framework are illustrated in Figure 4.

## 4 Methodology

### 4.1 Items generation

Item generation ensures the content validity of the scale (Churchill, 1979). Therefore, we developed an initial set of 125 Items based on the following three sources: (a) the focus group interviews, (b) previous research on TPB and NAT, and (c) previous research on green apparel purchase Behavior. Since the current study adopted mature scales from foreign research, in order to avoid interpretation biases caused by language and understanding differences. The foreign scales were translated in both directions by two master’s students and one international student and then reviewed and adjusted by the project team through discussions. The initial questionnaire underwent multiple trial fills and modifications to finally form the official questionnaire, which contains 12 measurement dimensions and 44 measurement items, as shown in Table 2.

**Table 2:** Measurement items of the questionnaire.

Variant	Coding	Entry	Sources
Behavioral Attitudes (BA)	BA1	Buying green apparel is a good idea.	Rausch and Kopplin (2021)
	BA2	Buying green apparel is a good decision.	
	BA3	Buying green apparel is an important thing.	
	BA4	I am a big supporter of buying green apparel.	
Subjective Norms (SN)	SN1	My purchase decisions are influenced by the perceptions and evaluations of those around me.	Yadav and Pathak (2016)
	SN2	I will buy clothing brands or products recognized and respected by those around me.	
	SN3	I will buy a clothing brand or product because it has received positive feedback from people around me.	
	SN4	I will not buy a clothing brand or product because it gets negative reviews from people around me.	
Perceived Behavioral Control (PBC)	PBC1	I will be controlled by my feelings and emotions when buying green apparel.	Yadav and Pathak (2016)
	PBC2	I can easily control my clothing purchases when buying green apparel.	
	PBC3	When buying green apparel, I can restrain my impulsive spending behavior and reasonably control my expenditure.	
Purchase Intention (PI)	PI1	I am willing to buy green apparel in the event of an environmental accident.	Rausch and Kopplin (2021)
	PI2	I am highly likely to buy green apparel in response to environmental accidents.	
	PI3	I am more likely to buy green apparel in response to environmental accidents.	
	PI4	I would recommend green apparel to others in the face of environmental accidents.	
Purchase Behavior (PB)	PB1	I will buy that green apparel more often in the future in the wake of environmental accidents.	Lao (2014)
	PB2	I am happy with the process of buying green apparel in the face of environmental accidents.	
	PB3	I am happy with the results of buying green apparel in the face of environmental accidents.	
	PB4	I buy green apparel quite frequently in response to environmental accidents.	
Awareness of consequences (AC)	AC1	I think consuming green apparel products is good for the environment.	Munerah et al. (2021)
	AC2	I feel that consuming green apparel products is good for the health of garment workers and those living in the area of origin.	
	AC3	I think producing environmentally unfriendly clothing causes severe pollution and environmental damage.	
Ascription of Responsibility (AR)	AR1	I believe that every consumer is responsible for the environmental problems caused by the production and consumption of clothing.	Joanes (2019)
	AR2	Consumers share some responsibility for the ecological and environmental problems caused by consuming non-green apparel products.	
	AR3	Consumers have some responsibility for the environmental problems caused by landfilling or incinerating discarded clothing.	
	AR4	As a consumer, you should take some responsibility for reducing clothing pollution.	
Personal Norms (PN)	PN1	I feel I must contribute to reducing the problem of clothing pollution.	Li and Shao (2023)
	PN2	We must all consider the environmental impact of clothing when we buy it.	
	PN3	My values will motivate me to choose to buy more eco-friendly clothing.	
	PN4	Over-consumption of non-environmentally friendly clothing makes me feel guilty.	
Scale of Environmental Accidents (ES)	ES1	The impact of this environmental accident extends beyond the geographic area in which it occurred.	FG
	ES2	The impact of this environmental accident extends beyond the industry in which it occurred.	
	ES3	This environmental accident affected a relatively large group of people.	
	ES4	The impact of this environmental accident was widespread.	
Effect Nature of Environmental Accidents (ENE)	ENE1	This environmental accident has strengthened my resolve to buy green apparel products.	FG
	ENE2	This environmental accident has drawn my attention to the natural environment.	
	ENE3	This environmental accident has made me take the environment more seriously.	
	ENE4	This environmental accident has led me to a better understanding of green apparel.	
Duration of Environmental Accidents (DE)	DE1	This environmental accident caused permanent damage to the environment.	FG
	DE2	It is unlikely that the environment in the area will improve over a long period.	
	DE3	The environmental impact of this environmental accident will be intensified.	
The Degree of Environmental Accidents’ Suddenness (DSE)	DSE1	This environmental accident came out of nowhere.	FG
	DSE2	There was no sign of this environmental accident before it happened.	
	DSE3	Neither brands nor consumers were prepared for this environmental accident before it happened.	

### 4.2 Sampling design and research tool

This study utilized a questionnaire survey method to randomly gather data from Generation Z consumers with purchase intentions and purchasing power. The data collection period was from June 2023 to July 2023. Data were collected using various strategies, including online survey platforms, social media, online forums, community groups, and offline surveys. Online survey platforms mainly choose Sojump (China’s largest online questionnaire platform) to distribute questionnaires, while social media, online forums mainly select Red Booklet, Weibo, and Zhihu to distribute questionnaires. Additionally, considering that the target audience for this research is Generation Z, which largely consists of college students, offline surveys were conducted at two university campuses and a streetwear clothing store. The timing and locations for the offline surveys were carefully selected to maximize foot traffic. The university campus surveys took place during the lunchtime period from 12:00 PM to 2:00 PM in the cafeteria, while the streetwear clothing store surveys were conducted on weekends from 10:00 AM to 5:00 PM at the store entrance. Both the online and offline questionnaire surveys offered participants the opportunity to enter a prize-based lottery after completing the questions. This approach was implemented to incentivize active participation and enhance the quality of responses. The goal was to gain a more precise understanding of how environmental accidents influence Generation Z’s purchasing behavior towards green apparel.

These methods and strategies ensured sample diversity and representativeness to understand Gen Z consumers’ purchase intentions and behaviors comprehensively.

Ethics considerations were also taken into account during the data collection process. Participants were informed of the purpose of the survey and its voluntary nature, and their responses were kept confidential. Informed consent was obtained before participation. This ensured the legality and ethicality of the survey.

We followed a widely used two-step approach (Talwar et al., 2020a,b) to analyze the data using SPSS 26.0 and AMOS 25.0. This approach involved conducting confirmatory factor analysis (CFA) to examine the measurement model, followed by structural equation modeling (SEM), as detailed in the next section. Prior to analysis, we thoroughly cleaned the data and addressed various issues. Out of the 588 collected questionnaires, 551 were included in the study for subsequent analysis after excluding invalid or incomplete questionnaires, with an effective proportion of 93.7%.

## 5 Results

The data collected in this study showed that female respondents accounted for 65.34%, possibly due to the higher frequency of apparel consumption among female consumers (Sadiq et al., 2021). Approximately 51.18% of the respondents were born between 1995 and 1999, followed by respondents born between 2000 and 2004, accounting for 48.82%. In addition, among the respondents, approximately 52.81% were undergraduates, followed by 24.68% of junior college students and 19.06% of graduate students (Table 3).

**Table 3:** Sample characteristics (*N* = 551).

Characteristic	Counts	Percent (%)
Gender		
Female	360	65.34
Male	191	34.66
Year of birth		
1995–1999	282	51.18
2000–2004	269	48.82
Educational background		
High school and below	19	3.45
Junior college	136	24.68
Undergraduate	291	52.18
Postgraduate and above	105	19.06
Monthly consumption level (RMB)		
0–2000	60	10.89
2000–4,000	242	43.92
4,000–10,000	178	32.3
10,000 and above	71	12.89
Careers		
Humanities and social sciences (journalists, writers, teachers, lawyers, politicians, etc.)	228	41.38
Science and technology (scientists, engineers, doctors, programmers, etc.)	190	34.48
Arts (painters, musicians, actors, designers, etc.)	133	24.14

### 5.1 Measurement model

This study conducted reliability and validity analyses on the questionnaire data using SPSS 26. Firstly, Cronbach’s Alpha coefficient was employed to examine the reliability of the questionnaire data. The Alpha coefficients for each variable are presented in Table 4. All variables exhibited Alpha coefficients above 0.7, and the overall Alpha value exceeded 0.9, indicating satisfactory reliability of the questionnaire data (EYSENCK and EYSENCK, 1968), as shown in Table 4.

**Table 4:** Results of the reliability test.

Constructs	Items	Cronbachs Alpha
Ensemble	36	0.940
Behavioral Attitudes (BA)	3	0.788
Subjective Norms (SN)	3	0.767
Perceived Behavioral Control (PBC)	3	0.804
Purchase Intention (PI)	3	0.827
Purchase Behavior (PB)	3	0.849
Awareness of Consequences (AC)	3	0.831
Ascription of Responsibility (AR)	3	0.813
Personal Norms (PN)	3	0.794
Scale of Environmental Accidents (ES)	3	0.827
Effect Nature of Environmental Accidents (ENE)	3	0.816
Duration of Environmental Accidents (DE)	3	0.795
The Degree of Environmental Accidents’ Suddenness (DSE)	3	0.765

Secondly, an assessment of content validity and construct validity was conducted to examine the extent to which the measurement results accurately reflect the intended content. Content validity primarily reflects the breadth and richness of the scale itself. The scales used in this study were developed based on multiple previously validated scales and focus group discussions (FG), ensuring content validity.

Prior to conducting exploratory analysis, a test of adequacy was performed to ensure the authenticity and effectiveness of the data. The Kaiser-Meyer-Olkin (KMO) value for the current data analysis was found to be 0.921, with an approximate chi-square value of 9680.684, degrees of freedom of 630, and a significance level of 0.000, indicating suitability for factor analysis (Balogh et al., 2001).

According to the results of Pearson correlation analysis in Table 5, the correlation coefficients between each independent variable and the dependent variable were all found to be significant at the 0.01 level, suggesting that the independent variables may have a positive impact on the dependent variable. A scale with good construct validity accurately reflects the characteristics of the targeted measurement and can be evaluated through convergent validity and discriminant validity. Convergent validity refers to the magnitude of the factor loading coefficients for each item reflecting the corresponding variable, often assessed through calculations of Average Variance Extracted (AVE) and Composite Reliability (CR). In confirmatory factor analysis, standardized factor loadings >0.5, AVE > 0.5, and CR > 0.7 demonstrate good convergent validity of the research data (Barrett, 2007). In this study, all variables exhibited significantly large standardized factor loadings (> 0.6), AVE values above 0.5, and CR values above 0.7, indicating satisfactory convergent validity of the research data. Additionally, the AVE values for each dimension were all above 0.5, indicating sufficient discriminant validity and shared variance among dimensions. The correlations among the twelve latent variables were below 0.90, suggesting that multicollinearity may not be an issue (Mason and Perreault, 1991).

**Table 5:** AVE values and squared correlation of each latent variable.

	BA	SN	PBC	PI	PN	AC	AR	PN	ES	ENE	DE	DSE
BA	**0.556**											
SN	0.479**	**0.532**										
PBC	0.327**	0.351**	**0.580**									
PI	0.418**	0.397**	0.532**	**0.615**								
PB	0.455**	0.362**	0.418**	0.472**	**0.850**							
AC	0.440**	0.371**	0.383**	0.432**	0.514**	**0.833**						
AR	0.401**	0.349**	0.395**	0.414**	0.410**	0.370**	**0.814**					
PN	0.444**	0.370**	0.331**	0.438**	0.455**	0.359**	0.571**	**0.797**				
ES	0.395**	0.349**	0.387**	0.444**	0.358**	0.392**	0.393**	0.338**	**0.829**			
ENE	0.405**	0.383**	0.422**	0.456**	0.408**	0.437**	0.414**	0.420**	0.556**	**0.817**		
DE	0.370**	0.340**	0.381**	0.433**	0.377**	0.335**	0.369**	0.342**	0.378**	0.398**	**0.795**	
DSE	0.342**	0.335**	0.340**	0.377**	0.353**	0.331**	0.350**	0.344**	0.322**	0.428**	0.463**	**0.763**

The average variance extracted (AVE) values are shown in bold text. The squared correlations (R2) of all constructs are on the off-diagonal. Structural model results. ** *p* < 0.01, and *** *p* < 0.001.

### 5.2 Structural model

**Figure 5:**
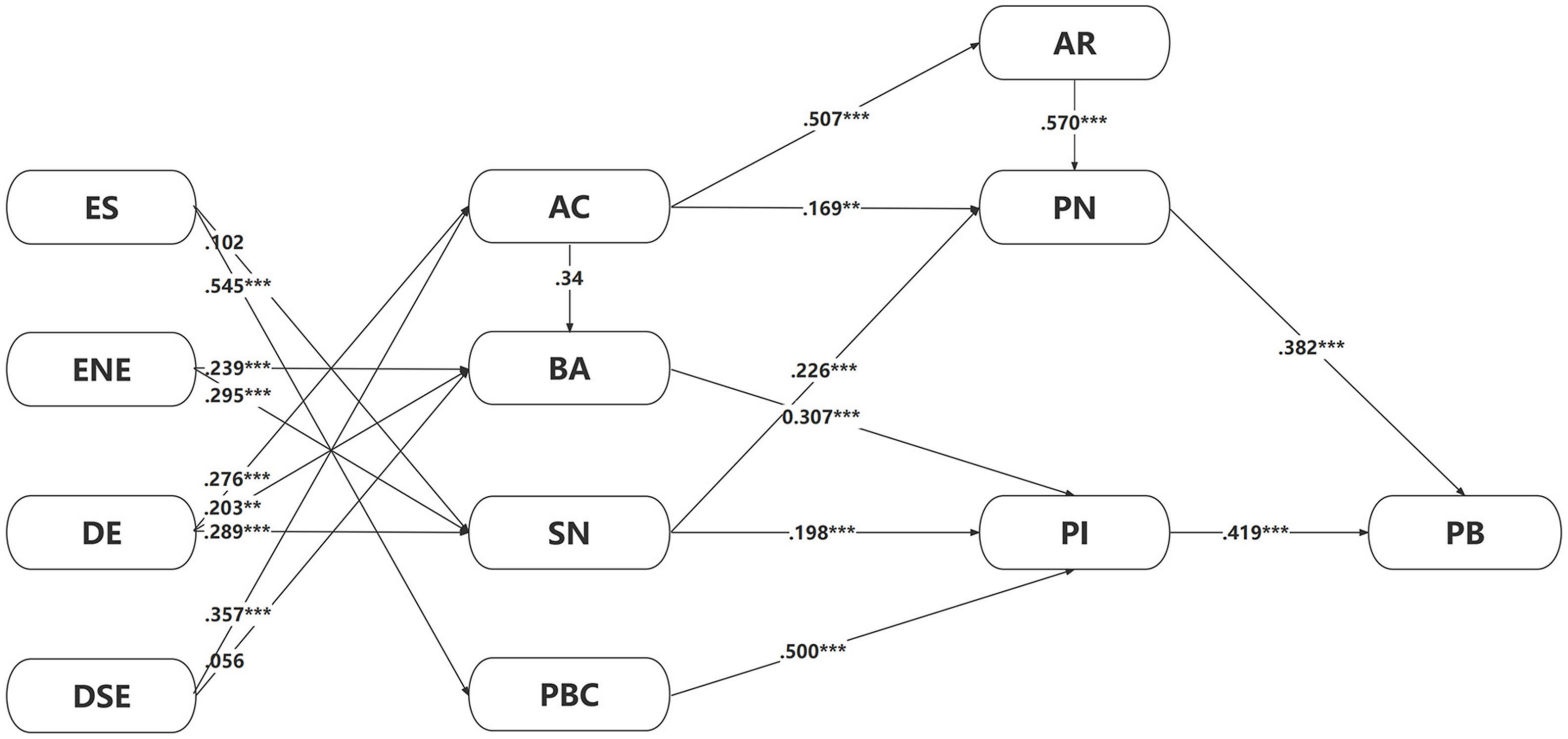
Structural equation modeling.

Similar to the measurement model, the structural model was also found to possess satisfactory model fit indices, as shown in Figure 5. The various indicators of model fit have all met the fitting standards, except for a slightly lower Goodness of Fit Index (GFI) (chi-square/df = 2.381, comparative fit index = 0.915, incremental fit index = 0.906, normed fit index = 0.708, relative fit index = 0.827, and root mean square error of approximation = 0.050), indicating a good fit for the model.

The significance of path coefficients in the model is reflected by critical ratios and *p*-values. A critical ratio absolute value greater than 1.96 indicates that the path coefficient has reached a significance level of 0.05. The results of the path analysis for the research model are shown in Table 6.

**Table 6:** Path analysis.

Suppose that	Trials	Estimate	SE.	CR.	*p*
H1	PI<---BA	0.307	0.055	5.46	***
H2	PI<---SN	0.198	0.062	3.496	***
H3	PI<---PBC	0.500	0.047	9.433	***
H4	PB<---PI	0.419	0.059	7.611	***
H5	AR<---AC	0.507	0.052	9.213	***
H6	PN<---AC	0.169	0.056	3.066	**
H7	PN<---AR	0.570	0.066	9.31	***
H8	PB<---PN	0.382	0.054	6.828	***
H9	BA<---AC	0.343	0.057	5.664	***
H10	PN<---SN	0.226	0.063	4.342	***
H11	SN<---ES	0.102	0.072	1.263	0.207
H12	PBC<---ES	0.545	0.059	10.157	***
H13	BA<---ENE	0.239	0.063	3.613	***
H14	SN<---ENE	0.295	0.071	3.534	***
H15	AC<---DE	0.276	0.077	3.791	***
H16	BA<---DE	0.203	0.069	2.967	**
H17	SN<---DE	0.289	0.059	4.374	***
H18	AC<---DSE	0.357	0.081	4.839	***
H19	BA<---DSE	0.056	0.078	0.749	0.454

***Means *p* less than 0.001; **means *p* less than 0.01; *means *p* less than 0.05.

According to Table 6, all hypotheses were verified and showed significant positive effects, except that the scale of the environmental accident had no significant impact on the subjective norms (H11), and the degree of Environmental Accidents’ suddenness had no significant impact on the behavioral attitudes (H19).

## 6 Discussion and implications

### 6.1 Discussion

This research examines Generation Zs green apparel purchase behavior by incorporating four dimensions of environmental accidents into the TPB and NAT. The research findings demonstrate that environmental accidents significantly impact Generation Zs green apparel purchase behavior.

Firstly, this research confirms that the TPB provides a solid theoretical basis for explaining Generation Z consumers green apparel purchase intentions in the apparel market. The research findings align with previous studies on the green consumption behavior of Generation Z (Kang et al., 2013; Rausch and Kopplin, 2021; Liu, 2022). The research reveals that the three factors of attitude, subjective norms, and perceived behavioral control in the TPB positively impact purchase intentions, which subsequently influence purchase behavior. Notably, this research also reveals that, among the influencing factors of Generation Z consumers green apparel purchase intentions, perceived behavioral control has a more significant impact than subjective norms and attitudes. This result is attributed to the fact that the actual purchase behavior of Generation Z consumers regarding green apparel reflects their intrinsic attitudes and standards and serves as an external expression of their habits and lifestyles (Dabija and Brandusa, 2017; Saarelainen, 2021). They tend to value autonomy and personal choice and prioritize the ability to control their behavior, focusing on personalization and self-expression (Statista, 2023). When purchasing green apparel, they consider various factors such as comfort, durability, and fashionable design, and perceived behavioral control plays a crucial role. Perceived behavioral control includes their awareness of the impact and significance of their green apparel purchase behavior (Djafarova and Foots, 2022) and their ability to control it. Perceived behavioral control includes their awareness of the impact of their purchase behavior and their ability to control it. It significantly influences purchase intentions and determines their actions. Purchasing green apparel is seen as both a consumption behavior and an environmental action for Generation Z. Attitudes and subjective norms establish a positive image of green consumption behavior and lead to positive psychological outcomes during the purchase process.

Furthermore, this study also confirms that NAT provides a solid theoretical foundation for explaining the green apparel purchase intentions of Generation Z. The research findings align with previous studies on the green consumption behavior of Generation Z (Schwartz, 1977; Ebreo et al., 2003; Schultz et al., 2005; Milfont et al., 2010; Chaturvedi et al., 2020). The study supports H5, H6, H7, H8, H9, and H10, indicating that the awareness of consequences, the ascription of responsibility, and personal norms are essential in Generation Z green apparel purchase intentions. Among these factors, the awareness of consequences has a more significant impact on the ascription of responsibility, and the ascription of responsibility considerably influences personal norms. This is because the awareness of consequences in NAT is recognized as an essential factor influencing individual behavioral motivation. At the same time, the ascription of responsibility represents the individuals identification of and adherence to social norms, and personal norms reflect the individuals awareness and internalization of social norms. The positive awareness of consequences and the ascription of responsibility demonstrated by Generation Z consumers in their green apparel purchase behavior reflect their commitment to environmental protection and adherence to social norms (Dabija and Brandusa, 2017, Saarelainen, 2021). The ascription of responsibility positively influences personal norms, indicating that when purchasing green apparel, Generation Z consumers value its eco-friendly attributes and prioritize their social responsibility and awareness of norms (Djafarova and Foots, 2022). Recognizing and internalizing this sense of social responsibility and awareness of norms strongly drives their green apparel purchase intentions.

Lastly, the research findings confirm support for H12, H13, H14, H15, H16, H17, and H18, indicating a solid interrelationship among the dynamic variables of environmental accidents, collectively influencing peoples reactions and behaviors towards these events. The uncertainty and panic generated by human beings in the face of environmental accidents lead to strong signaling effects in terms of the scale, nature of effects, and duration of these environmental accidents. Peoples perception of the scale of environmental accidents often leads to more robust emotional responses, motivating them to take action to control the development of these events (Van Bavel et al., 2020). The effect nature of environmental accidents primarily affects attitudes and subjective norms, as it relates to human emotional experiences. It affects individuals’ acceptance, reactions, and behavioral attitudes toward environmental accidents, and individuals typically follow certain behavioral norms and moral principles when faced with such events (Pyszczynski et al., 2003). This also reflects the influence of the effect nature of environmental accidents on personal norms. Therefore, Understanding the effect nature of environmental accidents helps in understanding individuals’ behavior and psychological states and in formulating response strategies.

Additionally, the duration of environmental accidents positively affects the awareness of consequences. The duration directly determines the disruptive period these events have on peoples lives, further influencing their judgment of the consequences of these events (Schultz and Stone, 1994; Avraham Bleich et al., 2003; Bhattacherjee and Sanford, 2006). On the other hand, the degree of environmental accidents’ suddenness also positively impacts the awareness of consequences. The suddenness represents unpredictability and unexpectedness. The increase in unpredictability and unexpectedness makes environmental accidents more likely to arouse individuals tension and concern, thus affecting their awareness of the consequences (Mileti, 1999).

Compared to other variables, the scale of environmental accidents significantly positively impacts perceived behavioral control, which can be attributed to the considerable sensory impact of event scale on humans. It can be seen as an alarm capable of triggering individuals nervous systems, thus influencing behavioral decision-making. The scale of environmental accidents can help individuals better understand the severity and make decisions earlier by providing more warning information (Van Bavel et al., 2020). Therefore, scientifically and effectively assessing the scale of environmental accidents and conducting timely and accurate information dissemination will help enhance individuals perceived behavioral control.

The data analysis of this research did not support two assumptions: H11 and H12. Firstly, the scale of environmental accidents did not significantly impact subjective norms in Generation Z’s green apparel purchase behavior. Secondly, the degree of Environmental Accidents’ suddenness did not considerably influence behavioral attitudes. However, the degree of Environmental Accidents’ suddenness did not considerably impact behavioral attitudes. This result may be because the influence of environmental accidents on subjective norms and behavioral attitudes is not direct but instead occurs through the cross-influence of factors such as individual identity, social values, and group interaction. In addition, it may be because Generation Z emphasizes personalization and self-expression in their consumption concepts and has high levels of concern about sustainability and environmental awareness (Chaturvedi et al., 2020; McCoy et al., 2021; Arora and Manchanda, 2022). These factors may outweigh the impact of environmental accidents on them, making their subjective norms and behavioral attitudes relatively independent of the effects of environmental accidents.

According to the results, the scale of environmental accidents significantly influences perceived behavioral control and perceived behavioral control significantly influences purchase intention. Meanwhile, purchase intention has a significant impact on purchase behavior, so compared with other variables, the scale of environmental accidents substantially affects Generation Z’s green apparel purchase behavior. In addition, the awareness of consequences has a significant impact on the ascription of responsibility, and the ascription of responsibility substantially affects personal norms. These results prove the critical practical value of NAT in studying green apparel purchase behavior.

### 6.2 Theoretical implications

This study integrates the Theory of Planned Behavior and the Norm Activation Theory to examine the impact of environmental accidents on Generation Z’s purchase behavior towards green apparel. Theoretical implications of this study are as follows:

Theoretical framework integration: This study combines the TPB and the NAT, which allows for a comprehensive understanding of the factors influencing Generation Z’s purchase behavior. By integrating these theories, this study expands the theoretical foundation regarding the impact of environmental accidents on consumer behavior, particularly within the context of green apparel.

Introduction of environmental accidents as variables: This study introduces environmental accidents as variables that influence Generation Z’s green apparel purchase behavior. By conceptualizing and measuring the dimensions of environmental accidents, such as scale, suddenness, nature of the effect, and duration, this study provides insights into the specific characteristics of environmental accidents and their influence on consumer behavior. This adds a new ideal to the study of environmental factors affecting purchase behavior.

Validation of the Norm Activation Theory: This study confirms the applicability of the NAT in examining Generation Z’s green apparel purchase behavior. By demonstrating that subjective norms play a significant role in shaping purchase intention, this study highlights the importance of social influence and normative considerations in the context of sustainable consumption. These findings contribute to the validation and further development of the NAT.

Value for research on environmental accidents: This study provides a theoretical foundation for studying environmental accidents by identifying specific dimensions that can be used to measure their impact on consumer behavior. The conceptualization of these dimensions can guide future research in examining the effects of different types of environmental accidents on various consumer behaviors, beyond the context of green apparel.

In conclusion, the theoretical implications of this study are noteworthy as they integrate two established theories, introduce environmental accidents as variables, validate the NAT, and provide a foundation for studying the impact of environmental accidents on consumer behavior. These implications contribute to the advancement of theoretical understanding in the field of sustainable consumption and provide valuable insights for future research on environmental accidents and consumer behavior.

### 6.3 Managerial implications

This study demonstrates that the scale, the degree of suddenness, nature of the effect, and duration of the environmental accident have varying degrees of influence on Generation Z’s awareness of consequences, behavioral attitudes, subjective norms, and perceived behavioral control toward green clothing purchases. Moreover, the scale of environmental accidents significantly impacts perceived behavioral control, which in turn affects Generation Z’s intention to purchase green apparel. Specifically, the following suggestions can be derived:

Enhance environmental awareness and strengthen green marketing: Companies should integrate efforts to enhance consumer awareness of environmental issues and implement effective green marketing strategies. By educating Generation Z consumers about the consequences of environmental accidents and promoting the ecological benefits of green apparel, companies can influence their purchase behavior and establish a competitive advantage in the market.

Improve product quality and transparency: Generation Z consumers are increasingly concerned about sustainability and ethical practices. Companies should ensure that their green apparel meets high-quality standards and adheres to transparent and ethical supply chain practices. By focusing on product quality and transparency, companies can build consumer trust and loyalty, thereby increasing their purchase behavior towards green apparel.

Collaborate with government and non-governmental organizations: Collaborations with government agencies and non-governmental organizations can be beneficial for companies in addressing the consequences of environmental accidents. These collaborations provide valuable resources, expertise, and networks to manage the repercussions of environmental accidents. Additionally, partnering with these organizations demonstrates the company’s commitment to sustainability and enhances its corporate social responsibility image.

Adjust business operations: Companies can utilize the theoretical model of this study to analyze the impact of environmental accidents on their own business and operations. Such analysis can help identify potential risks and take appropriate measures to mitigate them. Adjustments may include inventory management, procurement risk reduction, closer cooperation with suppliers, cost control, and increased production flexibility.

Understand consumer needs: The findings of this study provide insights into the green apparel purchase needs of Generation Z consumers. Companies can leverage these insights to gain a deeper understanding of consumer needs and accordingly adjust their product development and marketing strategies to align with consumer expectations. By meeting consumer expectations, companies can better cater to the green apparel purchase behavior of Generation Z.

By adopting these managerial suggestions, companies can navigate the challenges posed by environmental accidents and effectively address the impact on Generation Z’s green apparel purchase behavior. This not only enables companies to capture a larger share of the green apparel market but also demonstrates their commitment to social responsibility and sustainability.

## 7 Limitations and future research

This study has several limitations that should be acknowledged. Firstly, the data used in this study rely on self-reported measures, which may be subject to biases like social desirability bias and misrepresentation of purchase behavior. Secondly, the findings of this study are based on a sample of Generation Z consumers in a specific location, which may limit the generalizability of the results to other populations or contexts. Lastly, there may be other external factors, such as economic conditions and cultural values, that could influence the impact of environmental accidents on green apparel purchases but were not examined in this study.

Future research on the impact of environmental accidents on Generation Z’s purchase behavior towards green apparel can explore several directions. Firstly, studies can examine the effectiveness of interventions, like educational campaigns, in promoting green consumption among Generation Z. This would provide insights into strategies for businesses to enhance pro-environmental behavior. Secondly, further analysis should investigate the mediating effects of variables such as awareness of consequences, behavioral attitudes, and subjective norms on the relationship between environmental accidents and purchase intention. This would deepen our understanding of the underlying mechanisms driving purchase behavior. Thirdly, future studies should consider additional influencing factors like economic conditions, cultural values, and personal preferences in shaping green apparel purchase behavior. A comprehensive understanding of these various factors will aid businesses in tailoring their strategies accordingly. Lastly, it is crucial to investigate the intention-behavior gap and identify factors contributing to the inconsistency between green purchase intentions and actual behavior. This will help bridge the gap and promote sustainable consumption among Generation Z.

By addressing these limitations and further exploring the suggested research directions, future studies can contribute to a more robust and comprehensive understanding of the impact of environmental accidents on Generation Z’s purchase behavior towards green apparel.

## Data availability statement

The raw data supporting the conclusions of this article will be made available by the authors, without undue reservation.

## Ethics statement

Ethical approval was not required for the studies involving humans because the study on the psychology of purchasing green apparel and green apparel purchase behavior involving participants has been submitted to Zhejiang Sci-Tech University, China. The questionnaire’s introduction provides the research objectives, social value, scope of information collection, potential privacy risks, and measures taken to address them. Additionally, it includes the signatures and contact information of the researchers and the research institution. Participants are required to read the introduction to the questionnaire and then complete a written informed consent form. Based on this, the ethical risk of this study is extremely low and has obtained approval from Zhejiang Sci-Tech University, China. Therefore, there is no need for further ethical review. The studies were conducted in accordance with the local legislation and institutional requirements. The participants provided their written informed consent to participate in this study. Written informed consent was obtained from the individual(s) for the publication of any potentially identifiable images or data included in this article.

## Author contributions

LL: Writing – original draft, Validation, Formal analysis, Conceptualization. WZ: Writing – original draft, Visualization, Validation, Investigation, Conceptualization. HL: Writing – review & editing, Validation, Resources, Methodology, Investigation. ZZ: Writing – review & editing, Supervision, Software, Funding acquisition, Data curation.
